# Synthesis and Biological Evaluation of Novel Thiadiazole Derivatives as Antiplatelet Agents

**DOI:** 10.5812/ijpr-141846

**Published:** 2024-01-07

**Authors:** Mahsima Khakpash, Marjan Esfahanizadeh, Mohammad Mahboubi-Rabbani, Salimeh Amidi, Farzad Kobarfard

**Affiliations:** 1 Department of Medicinal Chemistry, School of Pharmacy, Shahid Beheshti University of Medical Sciences, Tehran, Iran; 2 Department of Medicinal Chemistry, Faculty of Pharmacy, Tehran Medical Sciences, Islamic Azad University, Tehran, Iran

**Keywords:** Thiadiazole, Antiplatelet, Arachidonic Acid (AA), Adenosine Diphosphate (ADP), Cyclization, P_2_Y_12_ Inhibitors

## Abstract

A novel series of thiadiazole compounds was synthesized through the reaction of thiosemicarbazone intermediates with 2, 3-dichloro-5,6-dicyano-1,4-benzoquinone (DDQ). The antiplatelet activity of the synthesized compounds was evaluated using an aggregation test with adenosine diphosphate (ADP) and arachidonic acid (AA) as platelet aggregation inducers. Among the synthesized analogs, compound 3b exhibited the most potent inhibition of platelet aggregation induced by ADP (half maximal inhibitory concentration [IC_50_] = 39 ± 11 µM). Molecular docking studies of 3b revealed hydrogen bonds between the nitrogen of the thiadiazole ring and Lys280. The tolyl ring exhibited hydrophobic interactions with Tyr105, similar to the antagonist co-crystallized with P_2_Y_12_ (PDB ID: 4NTJ). These compounds have the potential to serve as lead molecules for designing P_2_Y_12_ inhibitors.

## 1. Background

Cardiovascular disease (CVD), a leading global cause of death, is estimated to account for 17.5 million deaths (31%) annually (1). The prevalence of CVD and similar thrombotic diseases has been increasing (2, 3). Although platelets play a crucial role in preventing hemorrhage following injury, pathological platelet aggregation plays a significant role in CVDs and their complications (3). Therefore, one rational approach to prevent CVD is the use of antiplatelet drugs (4). As reported, antiplatelet drugs hold approximately a 65% market share and are the primary choice for preventing arterial thrombotic diseases (5).

Platelet activation can be triggered by exposure to potent endogenous stimuli, such as thrombin, thromboxane A_2_ (TXA_2_), collagen, and adenosine 5’-diphosphate (ADP) (6). Consequently, various types of antiplatelet drugs are available, each with a specific pharmacological mechanism (7). For instance, aspirin blocks the cyclooxygenase-1 (COX-1)/TXA_2_ pathway (8); nevertheless, others, such as clopidogrel and tirofiban, antagonize the P_2_Y_12_ ADP receptor and glycoprotein IIb/IIIa (GPIIb/IIIa), respectively (9). Phosphodiesterase inhibitors, such as cilostazol, belong to another class of antiplatelet agents that enhance the therapeutic efficacy of P_2_Y_12_ ADP receptor blockers (10).

Despite the advantages of antiplatelet drugs, their use is associated with some side effects, including gastrointestinal (GIT) disorders, drug resistance, and drug-drug interactions (4, 11). Despite significant progress in developing novel and efficient antiplatelet agents, there is still room for improving their efficacy and safety (12).

**Figure 1.**
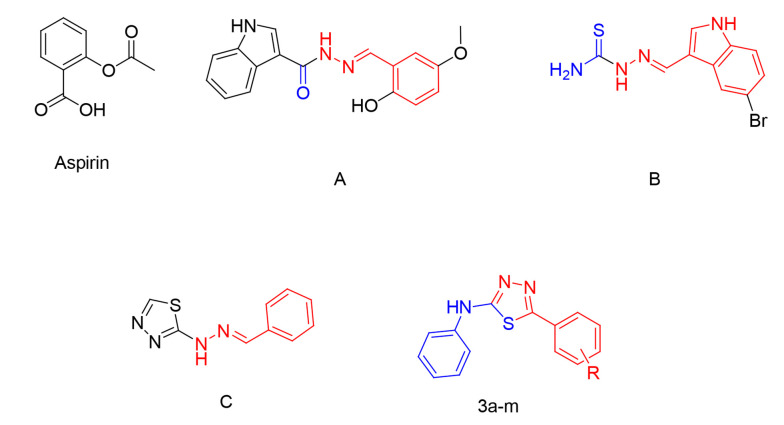
The chemical structures of A, B, C, and designed derivatives (3a-m)

In recent years, a diverse set of 1,3,4-thiadiazole analogs have been developed, exhibiting a broad spectrum of biological activities, such as antiparasitic (13), anticancer (14), antibacterial (15), antiviral (16), and antitubercular (17-19) activities. Furthermore, several studies on molecules with antiplatelet activity have revealed that analogs containing *N*-acylhydrazone and its isosteres exhibit remarkable antiplatelet activity (3, 20-23). Two examples of the compounds introduced in these studies, namely molecules A and B, are represented in Figure 1. 

In 1993, Rehse and Martens reported a novel series of 1,2,4-thiadiazolimines capable of blocking collagen-induced platelet aggregation at micromolar levels (24). Moreover, in one of our previous studies, a novel group of 2-hydrazinyl-1,3,4-thiadiazole analogs, exemplified by compound C (Figure 1), was synthesized with antiplatelet activity against arachidonic acid (AA) and ADP-induced platelet aggregation (25).

## 2. Objectives

The present study, aiming to discover new compounds with potent antiplatelet aggregation activity, synthesized a series of novel antiplatelet agents containing the thiadiazole moiety and screened their antiplatelet activity.

## 3. Experimental Section

### 3.1. General Methods

All reagents and solvents were procured from Merck (Darmstadt, Germany) and employed without additional purification. Proton nuclear magnetic resonance (^1^H-NMR) spectra were acquired using a 400 MHz Bruker spectrometer, with tetramethylsilane (TMS) as the internal standard. Chemical shifts were expressed as (δ = ppm), and CDCl_3_ and dimethyl sulfoxide-d_6_ (DMSO-d_6_) were utilized as solvents. Positive electrospray ionization (ESI) mass spectra were recorded on an Agilent 6410 triple quadrupole mass spectrometer. Infrared (IR) spectra were obtained using a Perkin Elmer IR spectrophotometer, with values reported in cm^-1^ and measured on potassium bromide discs. Melting points of the compounds were determined via the capillary method using an Electrothermal 9100 melting point apparatus, and the values provided were uncorrected. Thin layer chromatography (TLC) was conducted on silica gel (60) F_254_ Merck plates (Germany) and visualized under UV 254 nm light. Elemental analysis for carbon (C), hydrogen (H), nitrogen (N), and sulfur (S) was performed using a Costech 4010 elemental analyzer (Milan, Italy). For all the compounds, the calculated values closely matched the measured values within a margin of 0.4%.

#### 3.1.1. Synthesis of N-phenylhydrazinecarbothioamide (1)

Hydrazine hydrate (25 mmol, 0.125 g) was added to a solution of phenyl isothiocyanate (20 mmol, 0.27 g) in 20 mL of 2-propanol. The mixture was stirred for 4 hours at RT. The reaction progress was monitored by TLC. After the completion of the reaction, the resulting white precipitate was filtered and recrystallized from ethanol 96° to afford the purified intermediate 1.

White solid. Yield: (91%); mp: 138 - 141°C; IR (KBr): ϋ = 3300, 3149 (NH), 1216 (C=S) cm^-1^; ESI-MS m/z [M+H^+^] = 168.1, [M+Na^+^] = 190.1; Anal. Calcd for C_7_H_9_N_3_S: C, 50.27; H, 5.42; N, 25.13; S, 19.17, found: C, 51.31; H, 5.41; N, 25.14; S, 19.16.

#### 3.1.2. General Procedure for the Synthesis of Phenyl Thiosemicarbazone Analogs (2a-m)

The phenyl thiosemicarbazones 2a-m were synthesized via the procedure reported in the previous studies (26). In summary, a mixture of 1 (1 mmol) and appropriate aromatic aldehydes in absolute ethanol was stirred for 24 hours at RT. A catalytic increment of HCl (37%) was added to accelerate the reaction progress. The obtained precipitate was collected by filtration. The final products 2a-m were recrystallized from absolute ethanol. 

**(*E*)-2-benzylidene-*N*-phenylhydrazinecarbothioamide (2a): **White solid. Yield: (80 %); mp: 190 - 191°C; ^1^H-NMR (400 MHz, CDCl_3_): δ = 7.27 (t, J = 8.0 Hz, 1H, H4a), 7.44-7.40 (m, 5H, H3a, H5a, H3b, H5b,H4b), 7.69-7.66 (m, 4H, H2a, H6a, H2b, H6b), 7.98 (s, 1H, HC=N), 9.22 (s, 1H, NH), 10.34 (s, 1H, NH); IR (KBr): ϋ = 3296, 3150 (NH), 1582 (C=N), 1201 (C=S) cm^-1^; ESI-MS m/z [M+H^+^] = 256.0, [M+Na^+^] = 277.9; Anal. Calcd for C_14_H_13_N_3_S: C, 65.85; H, 5.13; N, 16.46; S, 12.56; found: C, 64.77; H, 5.12; N, 16.45; S, 12.57.

**(*E*)–2-(4-methylbenzylidene)-*N*-phenylhydrazinecarbothioamide (2b): **White solid. Yield: (86%); mp: 218 - 220°C; ^1^H-NMR (400 MHz, CDCl_3_): δ = 2.39 (s, 3H, CH_3_), 7.22 - 7.28 (m, 3H, HC4a, H3b, H5b), 7.42 (t, J = 8.0 Hz, 2H, H3a, H5a), 7.57 (d, J = 8.0 Hz, 2H, H2a, H6a), 7.67 (d, J = 8.0 Hz, 2H, H2b, H6b), 7.9 (s, 1H, HC=N), 9.20 (s, 1H, NH), 9.87 - 9.97 (m, 1H, NH); IR (KBr): ϋ = 3330, 3145 (NH), 1596 (C=N), 1199 (C=S) cm^-1^; ESI-MS m/z [M+H^+^] = 270.0, [M+Na^+^] = 291.8; Anal. Calcd for C_15_H_15_N_3_S: C, 66.88; H, 5.61; N, 15.60; S, 11.90; found: C, 65.22; H, 5.62; N, 15.62; S, 11.91.

**(*E*)-2-(4-bromobenzylidene)-*N*-phenylhydrazinecarbothioamide (2c): **White solid. Yield: (90 %); mp: 229-230°C; IR (KBr): ϋ= 3336, 3299 (NH), 1593 (C=N), 1265 (C=S) cm^-1^; ESI-MS m/z [M+H^+^] = 333.9, 335.9, [M+Na^+^] = 355.9, 357.9; Anal. Calcd for C_14_H_12_BrN_3_S: C, 50.31; H, 3.62; N, 12.57; S, 9.59; found: C, 51.02; H, 3.63; N, 12.56; S, 9.60.

**(*E*)-2-(2-chlorobenzylidene)-*N*-phenylhydrazinecarbothioamide (2d): **Cream-colored solid. Yield: (46%); mp: 188 - 189°C; ^1^H-NMR (400 MHz, CDCl_3_): δ = 7.24-7.44 (m, 6H, H3a, H4a, H5a, H3b, H4b, H5b), 7.68 (d, J = 8.0 Hz, 2H, H2a, H6a), 7.95 (dd, J = 8.0 Hz, J = 4.0 Hz, 1H, H6b), 8.30 (s, 1H, HC=N), 9.19 (s, 1H, NH), 9.48 (s, 1H, NH); IR (KBr): ϋ = 3287, 3136 (NH), 1591 (C=N), 1191 (C=S) cm^-1^; ESI-MS m/z [M+H^+^] = 289.9, [M+Na^+^] = 311.8; Anal. Calcd for C_14_H_12_ClN_3_S: C, 58.03; H, 4.17; N, 14.50; S, 11.07; found: C, 56.25; H, 4.16; N, 14.51; S, 11.06.

**(*E*)-2-(4-fluorobenzylidene)- *N*-phenylhydrazinecarbothioamide (2e): **White solid. Yield: (49%); mp: 199 - 200°C; ^1^H-NMR (400 MHz, CDCl_3_): δ = 7.12 (t, J = 8.0 Hz, 1H, H4a), 7.27 (t, J = 8.0 Hz, 2H, H3b, H5b), 7.42 (t, J = 8.0 Hz, 2H, H3a, H5a), 7.65 - 7.70 (m, 4H, H2a, H6a, H2b, H6b), 7.92 (s, 1H, HC=N), 9.16 (s, 1H, NH), 10.00 (s, 1H, NH); IR (KBr): ϋ= 3291, 3229 (NH), 1634 (C=N), 1235 (C=S) cm^-1^; ESI-MS m/z [M+H^+^] = 274; Anal. Calcd for C_14_H_12_FN_3_S: C, 61.52; H, 4.43; N, 15.37; S, 11.73; found: C, 60.53; H, 4.41; N, 15.36; S, 11.74.

**(*E*)-2-(4-methoxybenzylidene)-*N*-phenylhydrazinecarbothioamide (2f): **White solid. Yield: (52%); mp: 177 - 178°C; ^1^H-NMR (400 MHz, CDCl_3_): δ = 3.85 (s, 3H, OCH_3_), 7.26 (t, J = 8.0 Hz, 1H, H4a), 7.41 (t, J = 8.0 Hz, 2H, H3a, H5a), 7.62 (d, J = 8.0 Hz, 2H, H2a, H6a), 7.67 (d, J = 8.0 Hz, 2H, H2b, H6b), 7.92 (s, 1H, HC=N), 7.93 (d, J = 8.0 Hz, 2H, H3b, H5b), 9.19 (s, 1H, NH), 10.16 (s, 1H, NH); IR (KBr): ϋ = 3323, 3151 (NH), 1607 (C=N), 1205 (C=S) cm^-1^; ESI-MS m/z [M+H^+^] = 285.8; Anal. Calcd for C_15_H_15_N_3_OS: C, 63.13; H, 5.30; N, 14.73; S, 11.24; found: C, 62.98; H, 5.32; N, 14.71; S, 11.23. 

**(*E*)–2-(3-methoxybenzylidene)–*N*-phenylhydrazinecarbothioamide (2g): **Yellow solid. Yield: (70%); mp: 154 - 155°C; ^1^H-NMR (400 MHz, CDCl_3_): δ = 3.84 (s, 3H, OCH_3_), 6.96 (dd, J = 8.0 Hz, J=4 Hz, 1H, H4b), 7.20-7.34 (m, 4H, H4a, H2b, H5b, H6b), 7.42 (t, J = 8.0 Hz, 2H, H3a, H5a), 7.65 (d, J = 8.0 Hz, 2H, H2a, H6a), 7.97 (s, 1H, HC=N), 9.20 (s, 1H, NH), 10.64 (s, 1H, NH); IR (KBr): ϋ = 3327, 3150 (NH), 1596 (C=N), 1280 (C=S) cm^-1^; ESI-MS m/z [M+H^+^] = 285.9; Anal. Calcd for C_15_H_15_N_3_OS: C, 63.13; H, 5.30; N, 14.73; S, 11.24; found: C, 62.98; H, 5.32; N, 14.71; S, 11.23. 

**(*E*)-2-(4-chlorobenzylidene)-*N*-phenylhydrazinecarbothioamide (2h): **White solid. Yield: (57 %); mp: 199-200°C; IR (KBr): ϋ = 3302, 3122 (NH), 1587 (C=N), 1194 (C=S) cm^-1^; ESI-MS m/z [M+H^+^] = 289.8; Anal. Calcd for C_14_H_12_ClN_3_S: C, 58.03; H, 4.17; N, 14.50; S, 11.07; found: C, 57.33; H, 4.15; N, 14.52; S, 11.08.

**(*E*)-2-(3-cyanobenzylidene)-*N*-phenylhydrazinecarbothioamide (2i): **White solid. Yield: (63 %); mp: 197-198°C; IR (KBr): ϋ = 3285, 3177 (NH), 2237 (CN), 1542 (C=N), 1205 (C=S) cm^-1^; ESI-MS m/z [M+H^+^] = 281.0, [M+Na^+^] = 302.8; Anal. Calcd for C_15_H_12_N_4_S: C, 64.26; H, 4.31; N, 19.98; S, 11.44; found: C, 65.31; H, 4.30; N, 19.99; S, 11.45.

**(*E*)-2-(4-nitrobenzylidene)-*N*-phenylhydrazinecarbothioamide (2j): **White solid. Yield: (77 %); mp: 233-234°C; IR (KBr): ϋ = 3227, 3170 (NH), 1584 (C=N), 1521, 1339 (NO_2_), 1196 (C=S) cm^-1^; ESI-MS m/z [M+H^+^] = 300.8; Anal. Calcd for C_14_H_12_N_4_O_2_S: C, 55.99; H, 4.03; N, 18.65; S, 10.68; found: C, 55.50; H, 4.02; N, 18.63; S, 10.69.

**(*E*)–2-(3-hydroxybenzylidene)-*N*-phenylhydrazinecarbothioamide (2k): **White solid. Yield: (55 %); mp: 186-188°C; IR (KBr): ϋ = 3386, 3310 (NH), 1577 (C=N), 1161 (C=S) cm^-1^; ESI-MS m/z [M+H^+^] = 272.0; Anal. Calcd for C_14_H_13_N_3_OS: C, 61.97; H, 4.83; N, 15.49; S, 11.82; found: C, 60.71; H, 4.84; N, 15.50; S, 11.80.

**(*E*)-*N*-phenyl-2-(4-(trifluoromethyl)benzylidene) hydrazinecarbothioamide (2l): **White solid. Yield: (58.0 %); mp: 194-195 °C; IR (KBr): ϋ = 3200, 3060 (NH), 1609 (C=N), 1176 (C=S) cm^-1^; ESI-MS m/z [M+H^+^] = 324.2, [M+Na+] = 346.2; Anal. Calcd for C_15_H_12_F_3_N_3_S: C, 55.72; H, 3.74; N, 13.00; S, 9.92; found: 56.56; H, 3.72; N, 13.02; S, 9.93.

**(*E*)-2-(3-bromobenzylidene)-*N*-phenylhydrazinecarbothioamide (2m): **Yellow solid. Yield: (63 %); mp: 196-197°C; IR (KBr): ϋ = 3336, 3299 (NH), 1591 (C=N), 1214 (C=S) cm^-1^; ESI-MS m/z [M+H^+^] = 333.9, 335.9; Anal. Calcd for C_14_H_12_BrN_3_S: C, 50.31; H, 3.62; N, 12.57; S, 9.59; found: C, 50.36; H, 3.61; N, 12.55; S, 9.61.

#### 3.1.3. General Procedure for the Synthesis of Derivatives 3a-m

A solution of DDQ (0.53 g, 2.3 mmol) in acetonitrile was added dropwise to a solution of intermediates 2a-m (2.3 mmol) in the same solvent. The mixture was stirred in the RT overnight. The obtained precipitates 3a-m were filtered off and recrystallized from the absolute ethanol.

***N*-5-diphenyl-1,3,4-thiadiazol-2-amine (3a): **White solid. Yield: (75%); mp: 199 - 200°C; ^1^H-NMR (400 MHz, DMSO-d_6_): δ = 7.03 (t, 1H, J = 8.0 Hz, H4a), 7.37 (t, J = 8.0 Hz, 2H, H3a, H5a), 7.52 (m, 3H, H3b, H4b, H5b), 7.66 (d, J = 8.0 Hz, 2H, H2a, H6a), 7.87 (m, 2H, H2b, H6b), 10.56 (s, 1H, NH); IR (KBr): ϋ= 3252, 3198 (NH), 1610 (C=N), 695 (C-S) cm^-1^; ESI-MS m/z [M+H^+^] = 254.2; Anal. Calcd for: C_14_H_11_N_3_S: C, 66.38; H, 4.38; N, 16.59; S, 12.66; found: C, 65.42; H, 4.39; N, 16.58; S, 12.67.

***N*-phenyl-5-(*p*-tolyl)-1,3,4-thiadiazol-2-amine (3b): **White solid. Yield: (72%); mp: 219 - 220°C; ^1^H-NMR (400 MHz, DMSO-d_6_): δ = 2.37 (s, 3H, CH_3_), 7.02 (t, J = 8.0 Hz, 1H, H4a), 7.34 (m, 4H, H3b, H5b, H3a, H5a), 7.65 (d, J = 8.0 Hz, 2H, H2a, H6a), 7.75 (d, J = 8.0 Hz, 2H, H2b, H6b), 10.52 (s, 1H, NH); IR (KBr): ϋ = 3254, 3214 (NH), 1626, 1605 (C=N), 674 (C-S) cm^-1^; ESI-MS m/z [M+H^+^] = 268.2; Anal. Calcd for: C_15_H_13_N_3_S: C, 67.39; H, 4.90; N, 15.72; S, 11.99; found: C, 65.87; H, 4.91; N, 15.73; S, 11.98.

**5-(4-bromophenyl)-*N*-phenyl-1,3,4-thiadiazol-2-amine (3c): **White solid. Yield: (70%); mp: 230 - 231°C; ^1^H-NMR (400 MHz, DMSO-d_6_): δ = 7.03 (t, J = 7.6 Hz, 1H, H4a), 7.37 (t, 2H, J = 7.6 Hz, H3a, H5a), 7.66 (d, 2H, J = 7.6 Hz, H2a, H6a), 7.72 (d, 2H, J = 8.4 Hz, H3b, H5b), 7.82 (d, 2H, J = 8.8 Hz, H2b, H6b), 10.61 (s, 1H, NH); IR (KBr): ϋ = 3259, 3209 (NH), 1618, 1605 (C=N), 668 (C-S) cm^-1^; ESI-MS m/z [M+H^+^] = 332.1, 334.1; Anal. Calcd for: C_14_H_10_BrN_3_S: C, 50.62; H, 3.03; N, 12.65; S, 9.65; found: C, 51.55; H, 3.02; N, 12.67; S, 9.66.

**5-(2-chlorophenyl)-*N*-phenyl-1,3,4-thiadiazol-2-amine (3d): **White solid. Yield: (68%); mp: 225 - 226°C; ^1^H-NMR (400 MHz, DMSO-d_6_): δ = 7.04 (t, J = 8.0 Hz, 1H, H4a), 7.38 (t, J = 8.0 Hz, 2H, H3a, H5a), 7.53 (m, 2H, H4b, H5b), 7.67 (m, 3H, H2a, H6a, H6b), 8.09 (m, 1H, H3b), 10.59 (s, 1H, NH); IR (KBr): ϋ = 3261, 3203 (NH), 1622, 1604 (C=N), 671 (C-S) cm^-1^; ESI-MS m/z [M+H^+^] = 288.1; Anal. Calcd for: C_14_H_10_ClN_3_S: C, 58.43; H, 3.50; N, 14.60; S, 11.14; found: C, 56.59; H, 3.49; N, 14.61; S, 11.15.

**5-(4-fluorophenyl)-*N*-phenyl-1,3,4-thiadiazol-2-amine (3e): **White solid. Yield: (60%); mp: 199 - 200°C; ^1^H-NMR (400 MHz, DMSO-d_6_): δ = 7.03 (t, 1H, J = 7.2 Hz, H4a), 7.36 (m, 4H, H2a, H3a, H5a, H6a), 7.66 (d, J = 7.6 Hz, 2H, H3b, H5b), 7.92 (m, 2H, H2b, H6b), 10.56 (s, 1H, NH); IR (KBr): ϋ = 3291, 3229 (NH), 1634, 1614 (C=N), 683 (C-S) cm^-1^; ESI-MS m/z [M+H^+^] = 272.2; Anal. Calcd for: C_14_H_10_FN_3_S: C, 61.98; H, 3.72; N, 15.49; S, 11.82; found: C, 60.24; H, 3.71; N, 15.50; S, 11.81. 

**5-(4-methoxyphenyl)-*N*-phenyl-1,3,4-thiadiazol-2-amine (3f): **White solid. Yield: (68%); mp: 217 - 220°C; ^1^H-NMR (400 MHz, DMSO-d_6_): δ = 3.83 (s, 3H, OCH_3_), 7.01 (t, J = 8.0 Hz, 1H, H4a), 7.07 (d, J = 8.0 Hz, 2H, H3b, H5b), 7.36 (t, J = 8.0 Hz, 2H, H3a, H5a), 7.65 (d, J = 8.0 Hz, 2H, H2a, H6a), 7.8 (d, J = 8.0 Hz, 2H, H2b, H6b), 10.47 (s, 1H, NH); IR (KBr): ϋ = 3270, 3122 (NH), 1628, 1609 (C=N), 691 (C-S) cm^-1^; ESI-MS m/z [M+H^+^] = 283.8; Anal. Calcd for: C_15_H_13_N_3_OS: C, 63.58; H, 4.62; N, 14.83; S, 11.32; found: C, 62.78; H, 4.63; N, 14.82; S, 11.30.

**5-(3-methoxyphenyl)-*N*-phenyl-1,3,4-thiadiazol-2-amine (3g): **White solid. Yield: (71%); mp: 169 - 170°C; ^1^H-NMR (400 MHz, DMSO-d_6_): δ = 3.84 (s, 3H, OCH_3_), 7.05 (m, 2H, H4b, H4a), 7.41 (m, 5H, H3a, H5a, H2b, H5b, H6b), 7.66 (d, J = 8.0 Hz, 2H, H2a, H6a), 10.58 (s, 1H, NH); IR (KBr): ϋ = 3280, 3224 (NH), 1628, 1609 (C=N), 661 (C-S) cm^-1^; ESI-MS m/z [M-H-] = 282.2; Anal. Calcd for: C_15_H_13_N_3_OS: C, 63.58; H, 4.62; N, 14.83; S, 11.32; found: C, 64.20; H, 4.61; N, 14.80; S, 11.29.

**5-(4-chlorophenyl)-*N*-phenyl-1,3,4-thiadiazol-2-amine (3h): **White solid. Yield: (73%); mp: 119 - 220°C; ^1^H-NMR (400 MHz, DMSO-d_6_): δ = 7.04 (t, J = 8.0 Hz, 1H, H4a), 7.38 (t, J = 8.0 Hz, 2H, H3a, H5a), 7.58 (d, J = 8.0 Hz, 2H, H3b, H5b), 7.66 (d, J = 8.0 Hz, 2H, H2a, H6a), 7.89 (d, J = 8.0 Hz, 2H, H2b, H6b), 10.61 (s, 1H, NH); IR (KBr): ϋ = 3360, 3228 (NH), 1606, 1592 (C=N), 660 (C-S) cm^-1^; ESI-MS m/z [M-H-] = 286.1; Anal. Calcd for: C_14_H_10_ClN_3_S: C, 58.43; H, 3.50; N, 14.60; S, 11.14; found: C, 57.74; H, 3.51; N, 14.61; S, 11.15.

**3-(5-(phenylamino)-1,3,4-thiadiazol-2-yl)benzonitrile (3i): **White solid. Yield: (65%); mp: 219 - 220°C; ^1^H-NMR (400 MHz, DMSO-d_6_): δ = 7.049 (t, J = 7.6 Hz, 1H, H4a), 7.38 (t, J = 8.4 Hz, 2H, H3a, H5a), 7.67 (d, J = 8.0 Hz, 2H, H2a, H6a), 7.72 (t, J = 8.0 Hz, 1H, H5b), 7.96 (d, J = 8.0 Hz, 1H, H4b), 8.21 (d, J = 8.0 Hz,1H, H6b), 8.30 (s, 1H, H2b), 10.69 (s, 1H, NH); IR (KBr): ϋ = 3258, 3137 (NH), 2237 (CN), 1542, 1512 (C=N), 681 (C-S) cm^-1^; ESI-MS m/z [M+H^+^] = 279.1; Anal. Calcd for: C_15_H_10_N_4_S: C, 64.73; H, 3.62; N, 20.13; S, 11.52; found: C, 63.69; H, 3.61; N, 20.12; S, 11.53.

**5-(4-nitrophenyl)–*N*-phenyl-1,3,4-thiadiazol-2-amine (3j): **White solid. Yield: (68%); mp: 219 - 220°C; ^1^H-NMR (400 MHz, DMSO-d_6_): δ = 7.06 (t, 1H, J = 7.2 Hz, H4a), 7.39 (t, J = 7.6 Hz, 2H, H3a, H5a), 7.67 (d, J = 8.0 Hz, 2H, H2a, H6a), 8.12 (d, J = 8.4 Hz, 2H, H3b, H5b), 8.33 (d, J = 8.8 Hz, 2H, H2b, H6b), 10.78 (s, 1H, NH); IR (KBr): ϋ = 3332, 3106 (NH), 1589, 1570 (C=N), 1530, 1334 (NO_2_), 622 (C-S) cm^-1^; ESI-MS m/z [M+H^+^] = 299.1; Anal. Calcd for: C_14_H_10_N_4_O_2_S: C, 56.37; H, 3.38; N, 18.78; S, 10.75; found: C, 57.22; H, 3.37; N, 18.79; S, 10.74.

**3-(5-(phenylamino)-1,3,4-thiadiazol-2-yl)phenol (3k): **White solid. Yield: (81%); mp: 270 - 271°C; ^1^H-NMR (400 MHz, DMSO-d_6_): δ = 6.94 (d, J = 8.0 Hz, 1H, H4b), 7.08 (t, J = 8.0 Hz, 1H, H4a), 7.34 (m, 3H, H2b, H5b, H6b), 7.42 (t, J = 8.0 Hz, 2H, H3a, H5a), 7.71 (d, J = 8.0 Hz, 2H, H2a, H6a), 9.86 (s, 1H, NH), 10.59 (s, 1H, OH); IR (KBr): ϋ = 3233 (OH), 1594, 1569 (C=N), 681 (C-S) cm^-1^; ESI-MS m/z [M+H^+^ = 269.8; Anal. Calcd for: C_14_H_11_N_3_OS: C, 62.44; H, 4.12; N, 15.60; S, 11.90; found: C, 61.39; H, 4.11; N, 15.61; S, 11.92.

***N*-phenyl-5-(4-(trifluoromethyl)phenyl)-1,3,4-thiadiazol-2-amine (3l): **White solid. Yield: (65%); mp: 228 - 229°C; ^1^H-NMR (400 MHz, DMSO-d_6_): δ = 7.05 (t, J = 7.2 Hz, 1H, H4a), 7.39 (t, J = 8.0 Hz, 2H, H3a, H5a), 7.67 (d, J = 8.0 Hz, 2H, H2a, H6a), 7.87 (d, J = 8.4 Hz, 2H, H3b, H5b), 8.08 (d, J = 8.0 Hz, 2H, H2b, H6b), 10.70 (s, 1H, NH); IR (KBr): ϋ = 3253, 3199 (NH), 1610, 1598 (C=N), 660 (C-S) cm^-1^; ESI-MS m/z [M+H^+^] = 322.2; Anal. Calcd for: C_15_H_10_F_3_N_3_S: C, 56.07; H, 3.14; N, 13.08; S, 9.98; found: C, 55.96; H, 3.13; N, 13.07; S, 9.97.

**5-(3-bromophenyl)-*N*-phenyl-1,3,4-thiadiazol-2-amine (3m): **White solid. Yield: (67%); mp: 195 - 196°C; ^1^H-NMR (400 MHz, DMSO-d_6_): δ = 7.04 (t, J = 7.2 Hz, 1H, H4a), 7.38 (t, J = 8.4 Hz, 2H, H3a, H5a), 7.478 (t, J = 8.0 Hz, 1H, H5b), 7.70 (m, 3H, H2a, H6a, H4b), 7.86 (d, J = 8.0 Hz, 1H, H6b), 8.04 (s, 1H, H2b), 10.648 (s, 1H, NH); IR (KBr): ϋ = 3236, 3185 (NH), 1608, 1589 (C=N), 654 (C-S) cm^-1^; ESI-MS m/z [M+H^+^] = 332.0, 334.0; Anal. Calcd for: C_14_H_10_BrN_3_S: C, 50.62; H, 3.03; N, 12.65; S, 9.65; found: C, 51.52; H, 3.02; N, 12.64; S, 9.66.

### 3.2. Platelet Aggregation Studies

Platelet aggregation was evaluated using APACT-4004 aggregometer (LABiTec, Ahrensburg, Germany), based on the turbidimetric method reported by Born in previous studies (27). In summary, PRP was obtained through centrifugation of human-citrated blood at 100 g for 10 minutes. To obtain platelet-poor plasma (PPP), the residual blood was centrifuged at 1500 g for 15 minutes. Different concentrations of tested compounds were prepared in DMSO. Platelet-rich plasma (200 μL) and synthesized compounds were incubated at 37°C. After 5 minutes, the ADP and AA were added as platelet aggregation inducers. The final concentrations of ADP and AA were 5 μM and 1.35 mM, respectively. Dimethyl sulfoxide (0.5% v/v) was used as blank, and aspirin was used as positive control in the aggregation study. The platelet aggregation was monitored for 5 minutes. The compounds were screened at 1 mM. The active compounds of 3a-m (> 50% inhibition) were diluted to obtain IC50. The % inhibition values were obtained from the equation (28-31):


$$\%Inhibition=\left[1-\left(\frac{D}{S}\right)\right]\times 100$$

Where D denotes platelet aggregation in the presence of a tested molecule, and S stands for platelet aggregation in the presence of DMSO.

### 3.3. Docking Studies

The crystal structure of COX-1 (PDB code: 3N8Y) and purinergic receptor P_2_Y_12_ (PDB code: 4NTJ) were retrieved from the RCSB Protein Data Bank with a resolution of 2.60 and 2.62 Å, respectively (32, 33). The computer simulation automated docking analysis was performed with AutoDock Vina (34). The target protein was prepared by removing co-crystallized ligands and water molecules and adding the polar hydrogens and Gasteiger partial charges. The chemical structures of newly designed analogs were sketched and optimized using the molecular mechanic AMBER method with the algorithm Polak-Ribiere through Hyperchem 8.0 software (Gainesville, FL, USA). The central zones of the co-crystallized ligands in the active sites were determined as grid box’s centroids. The type of amino acid in the target protein that was involved in the formation of H-bond (distance < 3 Å) was predicted using Discovery Studio 4.5 visualizer (35). The final results were shown using PyMOL (36) and the ProteinsPlus web server (https://proteins.plus) (37).

## 4. Results and Discussion

### 4.1. Chemistry

**Figure 2.**
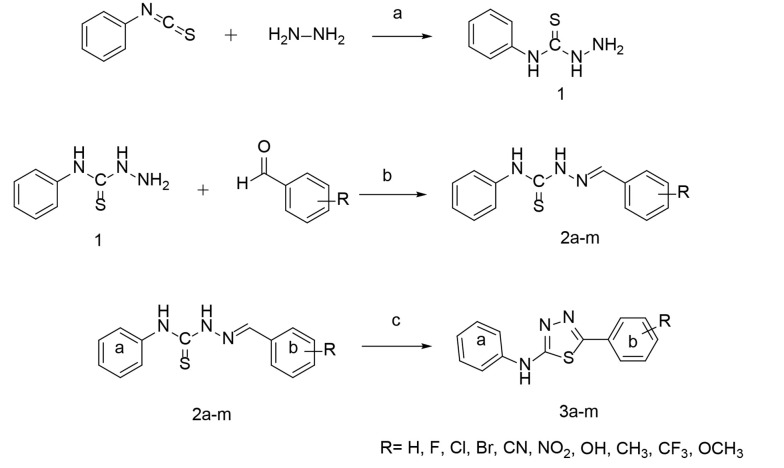
Synthetic route for the target compounds; reagents and conditions: (A), 2-propanol, RT; (B), HCl 37%, ethanol, RT; (C), acetonitrile, DDQ, RT

The newly designed compounds 2a-m were synthesized by reacting thiosemicarbazide intermediate 1 with appropriate benzaldehyde derivatives. Thiosemicarbazone derivatives 2a-m, therefore obtained, were subsequently converted to compounds 3a-m in acetonitrile at room temperature (RT), following the synthetic route outlined in Figure 2. Compound 1 was synthesized by adding 1 mmol of hydrazine hydrate to a solution of phenyl isothiocyanate in 2-propanol at RT. The chemical structure of the product was confirmed through infrared (IR) spectroscopy, as evidenced by the appearance of the N-H band at approximately 3300 cm^-1^. Compounds 2a-m resulted from the reaction of 1 mmol of phenyl thiosemicarbazide 1 with 1 mmol of an ethanolic solution of the corresponding benzaldehyde analogs in the presence of hydrochloric acid (HCl) as the catalyst. The primary IR characterization was indicated by the appearance of the C=N peak at 1550-1600 cm^-1^. Compounds 3a-m were prepared by stirring the solution of thiosemicarbazones 2a-m in acetonitrile with 2,3-dichloro-5,6-dicyano-1,4-benzoquinone (DDQ) for 2 hours. The IR spectrum was characterized by a distinct peak for N-H in the range of 3100-3400 cm^-1^.

Proton nuclear magnetic resonance (^1^H-NMR) spectroscopy displayed characteristic signals for the prepared compounds (3a-m). Compound 3a exhibited a distinct singlet peak at δ = 10.56 ppm, which is attributed to the N-H group, indicating aminothiadiazole formation. Compound 3b recorded a singlet peak at δ = 2.37 ppm for C-H protons originating from the aliphatic (CH_3_) group. In the ^1^H-NMR spectra of compounds 3f and 3g, significant singlet peaks appeared at δ = 3.83 and 3.84 ppm, integrating for three protons, indicating the presence of aliphatic methoxy groups (OCH_3_). Compound 3k displayed one prominent signal at δ = 10.59 ppm, attributed to the OH group. All corresponding aromatic protons for compounds 3a-m were observed in their expected aromatic region.

### 4.2. Antiplatelet Activity

**Figure 3.**
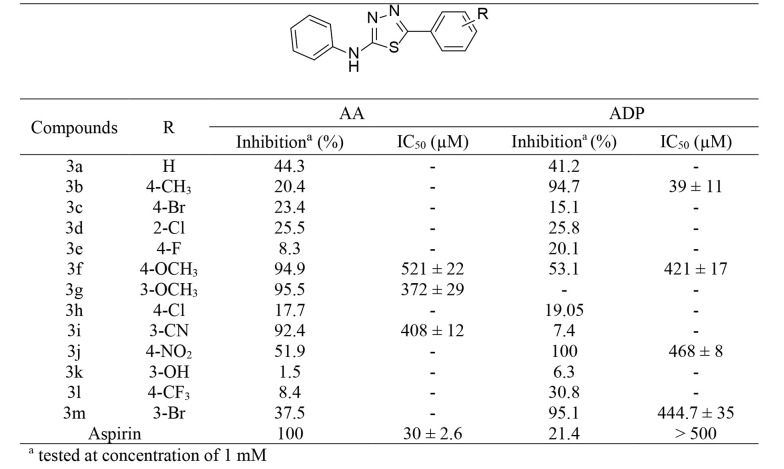
Inhibition percentage (%) and IC50 values (mean ± standard error of the mean [SEM], n = 3) of compounds (3a-m), using ADP and AA as platelet aggregation inducer agents

The antiplatelet activity of the synthesized compounds was evaluated using the “Born” method with ADP and AA as platelet aggregation inducers, with aspirin used as a positive control. The antiplatelet activity and half maximal inhibitory concentration (IC_50_) values of 3a-m are reported in Figure 3. 

The antiplatelet activity of 2a-m was evaluated at a concentration of 1 mM. All tested compounds exhibited less than 60% inhibition of platelet aggregation when ADP was used as the platelet aggregation inducer. When AA was used as a platelet aggregation inducer, only 2a, 2e, 2f, and 2k showed inhibition of more than 80%. The results obtained indicated that 2a-m did not possess suitable antiplatelet activity.

On the other hand, among the final products 3a-m, compounds 3f, 3g, and 3i, containing a 4-methoxy, 3-methoxy, and 3-cyano group, respectively, showed moderate effects against platelet aggregation induced by AA with IC_50_ values of 370 - 520 µM. Meanwhile, aggregation induced by the ADP molecule was more effectively inhibited by compounds 3f, 3j, and 3m. These compounds, with 4-methoxy, 4-nitro, and 3-bromo moieties, exhibited modest activity against platelet aggregation induced by ADP (IC_50_ = 421 - 468 μM). Among the cyclic compounds, 3b, containing 4-methyl with an IC_50_ of 39 ± 11 µM, was observed to be the most potent against ADP.

Thiosemicarbazones were converted into thiadiazole derivatives to study the effect of the ring on antiplatelet activity. According to the obtained results, the antiplatelet activity against aggregation induced by AA decreased after the ring closure of thiosemicarbazones; nevertheless, greater activity was observed against ADP-induced platelet aggregation with thiadiazole derivatives. Moreover, the addition of an electron-donating group, such as a methyl group at the para position of the phenyl ring (3b), significantly improved the antiplatelet activity against the ADP pathway; however, the introduction of electron-withdrawing groups, such as fluorine at the same position, had the opposite effect.

In addition, for compounds 3f and 3g, placing the methoxy group on the phenyl ring, regardless of the position of the substitution, increased the antiplatelet effect against AA-induced platelet aggregation.

### 4.3. Lipinski’s Rule of Five

To predict the oral suitability of the chemical compounds, the most active derivatives against ADP (3b) and AA (3f, 3g, and 3i) were evaluated in silico using a computational cheminformatics pipeline, namely Lipinski’s Rule of Five, employing SwissADME^®^ (38-40). LogP is one of the components of Lipinski’s Rule of Five and describes the lipophilicity of molecules. According to the calculated logP values, the compounds were predicted to be capable of crossing biological membranes due to their high lipophilicity. The results of calculating Lipinski’s criteria of drug-likeness for the most active compounds (Table 1) showed that none of them violated Lipinski’s boundaries, thereby suggesting a proper kinetic profile and good oral bioavailability.

**Table 1.** In Silico Predicted ADME Parameters of the Most Active Derivatives

Compounds	MW	HBD	HBA	LogP	Violation of Lipinski’s Rule of Five ^a^
**3b**	267.35	1	2	3.78	0
**3f**	283.35	1	3	3.43	0
**3g**	283.35	1	3	3.43	0
**3i**	278.33	1	3	3.21	0

^a^ Lipinski’s Rule of Five: Molecular weight (MW) ≤ 500 Da; Number of hydrogen bond donors (HBD) ≤ 5, number of hydrogen bond acceptors (HBA) ≤ 10, and logP ≤ 5.

### 4.4. Docking Studies

**Figure 4.**
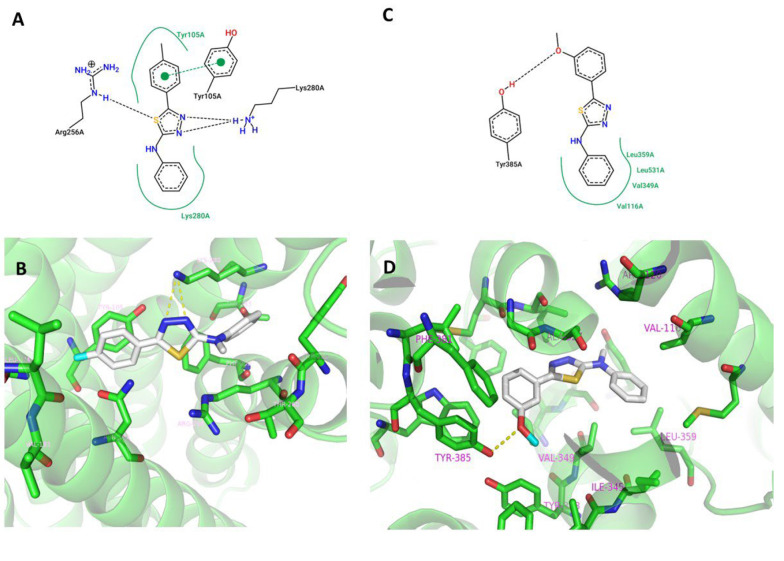
2D (A) and 3D (B) representation of 3b in the active site of P_2_Y_12_ (PDB ID: 4NTJ) and 2D (C) and 3D (D) representation of 3g in the active site of COX-1 (PDB ID: 3N8Y); Hydrogen bonds are represented as black and yellow dashed lines in 2D and 3D models respectively.

Based on the results of the antiplatelet aggregation test, compounds 3b and 3g, which exhibited the highest potency against ADP and AA-induced platelet aggregation, respectively, were selected for molecular docking simulations to model their binding to potential targets, namely P_2_Y_12_ and COX-1. The best binding poses with the lowest energy were calculated using AutoDock Vina and analyzed using PyMol and Discovery Studio Visualizer software. The binding modes of compounds 3b and 3g in the active sites of P_2_Y_12_ and COX-1 are depicted in Figure 4. 

The results suggest that in the case of 3b, P_2_Y_12_ aids in anchoring the compound within the pocket through hydrophobic interactions formed between the tolyl and phenyl rings in the ligand and Tyr105 and Lys280 in P_2_Y_12_, respectively. Additionally, π-π stacking occurs between the phenol ring of Tyr105 and the *p*-tolyl group of 3b. Moreover, hydrogen bonds are formed between Lys280 and the thiadiazole ring. These interactions are consistent with previously reported studies (41).

In the case of compound 3g, a hydrogen bond is observed between the hydrogen of the OH group of Tyr385 and the oxygen of the methoxy group. This hydrogen bond is also reported for diclofenac, the co-crystallized ligand, in the active site of COX-1. Furthermore, the phenyl ring of 3g forms hydrophobic interactions with Leu359, Leu531, Val349, and Val116 (32).

## 5. Conclusions 

In the present study, a novel series of thiadiazole derivatives were synthesized, and their activity on platelet-rich plasma (PRP) was evaluated using ADP and AA as platelet aggregation inducers. The synthesized compounds were thoroughly characterized through ^1^H-NMR, mass spectrometry (MS), IR, and elemental analyses. The findings revealed that most of the synthesized analogs exhibited low to moderate activity against AA and ADP. Structure-activity relationship (SAR) studies indicated that compound 3b, bearing a 4-methyl substituent on the aryl side group, displayed the highest potency against ADP (IC_50_ = 39 ± 11 µM). On the other hand, for antiplatelet activity against AA, derivatives containing substituents, such as 4-methoxy, 3-methoxy, and 3-cyano, exhibited the most activity.

In addition, in silico ADME prediction studies, the synthesized compounds adhere to Lipinski's Rule of Five parameters, suggesting their potential for oral administration. Furthermore, molecular docking analysis was conducted for the potent compounds 3b and 3g to study their putative binding patterns at the P_2_Y_12_ and COX-1 active sites, respectively. Overall, the presence of thiadiazole in these compounds was observed to potentiate their antiplatelet activity against ADP-induced aggregation. In summary, the results of this study provide valuable insights for future investigations aimed at developing potent compounds with antiplatelet effects.

## Data Availability

The dataset presented in the study is available on request from the corresponding author during submission or after publication.
